# Dealing with a Crisis: Does Covid-19 Promote Traditional Gender Roles?

**DOI:** 10.5334/pb.1032

**Published:** 2021-07-23

**Authors:** Alyson Sicard, Sandrine Redersdorff, Céline Darnon, Delphine Martinot

**Affiliations:** 1 Laboratoire de Psychologie Sociale et Cognitive (LAPSCO), CNRS UMR 6024, Université Clermont Auvergne, Clermont-Ferrand, France

**Keywords:** gender, Covid-19, citizenship behaviors, altruism, sacrifice

## Abstract

The Covid-19 crisis has many characteristics susceptible to emphasize gendered prescriptions. In the present research, we argue that the Covid-19 crisis should promote citizenship behaviors (CB) consistent with gender stereotypes. Two pre-registered experiments were conducted during lockdown in France (Study 1) and United Kingdom (Study 2). We manipulated the salience of the Covid-19 crisis using a fake newspaper article and showed that women were more likely than men to engage in CB of altruism and sacrifice. Meta-analysis results of the two studies confirmed that these gender differences were larger when the Covid-19 crisis was highly salient (vs. control condition). For women, more than for men, engaging in altruistic behaviors and making sacrifice for the greater good are perceived as the behaviors to endorse to cope with the Covid-19 crisis.

As the coronavirus has plunged the world into an unprecedented crisis, several voices are rising to signal its potential impact on gender equality. Indeed, the pandemic affects men and women differently. Women, for instance, are overrepresented among health and social workers, which are particularly exposed to the virus and the marginalization associated with this exposition (UNFPA, 2020). Women are also the primary victims of domestic violence, for which the risk is higher during lockdown (UN Women, 2020a), and the first affected by the restriction of access to sexual and reproductive healthcare. The United Nations therefore urges governments to take into account gender equality and women’s rights in their response to the Covid-19 crisis (United Nations Department of Global Communications, 2020). However, the role of gender in individual responses to the crisis remains quite unexplored so far. Do men and women react differently to this extraordinary situation?

To examine this question, we focused on men’s and women’s attitudes towards citizenship behaviors (CB), a concept originally developed in organizational psychology that has since been applied to other domains such as education (e.g., Meriac, 2012) or sport (Aoyagi et al., 2008). Organizational CB refer to “individual behavior that is discretionary, not directly or explicitly recognized by the formal reward system and that in the aggregate promotes the effective functioning of the organization” (Organ, 1998, p. 4). Altruism, sportsmanship, individual initiative and civic virtue are among the different forms of CB identified in the literature (e.g., Lepine et al., 2002; Podsakoff et al., 2000). In this research, we apply the concept of CB to society in general and its functioning in a time of crisis to investigate discretionary behaviors that promote the proper functioning of the society (rather than that of the organization) in the context of the Covid-19 epidemic but are not associated with direct and concrete rewards. We think CB are particularly relevant in the context of the Covid-19 crisis. Indeed, throughout the Covid-19 crisis, people were frequently encouraged to be “team players” and have had the opportunity to adopt a variety of behaviors supposed to facilitate national or international crisis management, like looking after their loved-ones, wearing a mask, or continuing to work from home (and sometimes outside of home). The objective of the present research is therefore to examine whether men and women differ or not in the type of CB they adopt in response to the Covid-19 crisis, and the extent to which they are likely to engage in these behaviors.

Gender stereotypes tell men and women how they should be and behave by prescribing them different traits and behaviors. Women are expected to be warm, interested in children and sensitive, whereas men are expected to be self-reliant, ambitious and assertive (Prentice & Carranza, 2002). Research suggests that the type of CB, and more generally the type of prosocial behaviors, men and women engage in are, in part, determined by those gendered prescriptions (Allen & Jang, 2018; Eagly, 2009; Kidder, 2002). Women are expected to show more altruism and sportsmanship than men, who are expected to show more civic virtue and individual initiative than women (Chiaburu, Sawyer, et al., 2014; Clarke & Sulsky, 2017; Heilman & Chen, 2005). Indeed, altruism (e.g., helping others) and sportsmanship (e.g., tolerating inconvenience without complaining) are consistent with the characteristics stereotypically prescribed to women, such as warm, caring and polite (Prentice & Carranza, 2002). Conversely, individual initiative (e.g., going beyond what is minimally required) and civic virtue (i.e., monitoring threat and opportunities) are consistent with stereotypes portraying men as assertive and independent. People usually act in accordance with those prescriptions and expectations as men and women differ in the type of help and support they offer in the workplace (i.e., organizational CB) and in other settings (e.g., close relationships; Allen & Jang, 2018; Eagly, 2009; Kidder, 2002). Men are more likely to perform agentic behaviors such as taking the initiative to help a stranger or providing collective support that promotes the interest of organizations and nations at war. Women, however, tend to opt for communal actions by caring and supporting relatives, friends and colleagues and engaging in community volunteering (Allen & Jang, 2018; Eagly, 2009). Thus, we can expect that, regardless of the context, men would report more CB of civic virtue and individual initiative than women, who would report more altruism and sportsmanship than men.

In addition, CB seem to be directly related to system justification and system threat. System justification theory (Jost & Banaji, 1994) posits that people are motivated to defend and rationalize existing social arrangements, even at the expense of personal and group interests. Such motivation is known to be influenced by context (Friesen et al., 2019; Jost, 2019). Chiaburu, Harris, et al., (2014) demonstrated that people expected more sportsmanship from women following an experimental manipulation of system threat (vs. a control, no threat condition). Moreover, they showed that system justification (which often occurs when the system is threatened) is positively correlated to expectations of sportsmanship from women (but not from men) in that the more people endorsed system justification beliefs, the more they expected women to engage in sportsmanship. Several of the contextual features susceptible to enhance people’s motivation to support the system are present in the Covid-19 crisis. Indeed, the current crisis comes with threats to national systems (e.g., high risk of economic crisis), dependence on the system (e.g., for financial assistance or protection against the virus), feeling of inescapability (the virus has spread all over the world) and low personal control (e.g., stay-at-home order; Al Dhaheri et al., 2021; Goodwin et al., 2021; Xiong et al., 2020), which are all contextual factors identified as promoting system justification (Friesen et al., 2019; Jost, 2019; Kay & Friesen, 2011). Consequently, people’s motivation to defend the existing system should be particularly high when they are led to think of the Covid-19 crisis.

As system justification is associated with traditional ideologies regarding social arrangements, including gender relations (Friesen et al., 2019; Jost & Hunyady, 2005), we argue that making this threat to the system salient would promote traditional gender roles. More specifically, the Covid-19 crisis could lead men and women to react in a way that is consistent with gender stereotypes and could therefore emphasize the tendency of men and women to engage in different types of CB. We therefore hypothesize that the stereotypical gender differences in CB described above would be more important when the Covid-19 crisis is salient (high salience condition) compared to a control condition (low salience of the Covid-19 crisis condition). More precisely, in such a condition of system threat, men should report more CB of civic virtue and individual initiative than women, who should report more altruism and sportsmanship than men. For similar reasons, the Covid-19 crisis could also promote traditional gender attitudes. Indeed, research has shown that the motivation to protect the system is associated with increased endorsement of the committed relationship ideology (Day et al., 2011), greater anti-feminist backlash (Yeung et al., 2014), greater blaming of a rape victim (Ståhl et al., 2010) and greater endorsement of essentialist explanations for gender differences (Brescoll et al., 2013). It can also trigger self-stereotyping (Bonnot & Jost, 2014; Laurin et al., 2011). Laurin et al. (2011) demonstrated that the motivation to justify the inequality causes men and women to align with complementary gender stereotypes. Women and men respectively rate themselves as more communal and more agentic when confronted with salient inequality (compared to a control condition). We therefore hypothesize men and women to feel more pressured to conform to the prescriptions of gender stereotypes when the Covid-19 crisis is salient compared to a control condition.

## The present research

These hypotheses were tested in two similar studies. In April 2020, we conducted a first study (Study 1) among French people under lockdown. To increase confidence in our findings, we decided to carry out a replication of Study 1 in another sample (Asendorpf et al., 2013; Brandt et al., 2014; Świątkowski & Dompnier, 2017). Study 2 was therefore conducted in May 2020 with British participants. Our choice to go from a French to a British sample was founded on two arguments. First, replicating our study in a different population increases the generalizability of our findings (if replicated). Second, by the time we conducted Study 2, France had eased the lockdown restrictions (after eight weeks of complete lockdown) and initiated a gradual return to normal. We could hardly anticipate or control the impact of such change on participants’ perspective. Great Britain, however, retained the stay-at-home order at this time (except for England, which was therefore not included in the study). Great Britain therefore provided a more suitable setting for Study 2 to closely replicate Study 1. Both studies were preregistered on Open Science Framework (OSF; *https://osf.io/gme2u/registrations*) and the ethics committee of the university approved their conduct (IRB00011540-2020-38). All material and data are also available through the OSF link.

## Method

### Participants

Since no previous research had addressed a similar issue, we conducted our a-priori power analysis based on a small-sized effect (f = 0.10). These analyses suggested that a minimum of 300 participants was required to detect an effect of this size with a repeated-measure ANOVA (cf. pre-registrations for the details). We planned to slightly oversample to anticipate the exclusion of some participants based on pre-established criteria. Participants were paid around 3 euros in Study 1 and 1.4 euros for Study 2.^1^

#### Study 1

Three hundred and seventy-seven participants accessed the online questionnaire. Once we excluded the participants who did not complete the questionnaire entirely (n = 19), those who failed the attention checks (n = 10), those with an aberrant completion time (+/– 3 SD; n = 6), as well as two non-French individuals (who reported living in France for only a year), our final sample included 340 French adults, with 189 men and 151 women. The participant’s age ranged from 18 to 78 years old, with a mean of 32.26 years old (*SD* = 13.20).

#### Study 2

Among the 367 people who answered the questionnaire, we excluded 7 participants who did not complete it entirely, 32 who failed attention checks and five who had an aberrant completion time (+/– 3 SD). Our final sample therefore comprised 323 British residents from Wales, Northern Ireland and Scotland, with 110 men and 213 women. The participant’s age ranged from 18 to 72 years old, with a mean of 35.96 years old (*SD* = 13.20).

### Procedure

The study was presented as a research on citizenship behaviors. After giving their online consent, participants were randomly assigned to one of the two conditions of the Covid-19 salience (high salience vs. control condition). Participants in the high-salience condition read a (fake but realistic) newspaper article on the current Covid-19 crisis, and subsequently completed the manipulation check measure. Participants in the control condition did not see the article and started by completing directly the measures. Participants of this condition filled out the manipulation check items at the end of the survey to avoid enhancing the salience of the crisis. Then, all participants completed measures assessing their system justification beliefs, the prescriptions of gender stereotypes and their likelihood to engage in CB.^2^

### Measures

#### Manipulation check

Participants were asked to indicate how worried they were about the Covid-19 crisis on a continuous response scale ranging from 0 (*not worried at all*) to 100 (*extremely worried*).

#### Citizenship behaviors

Based on the Organizational Citizenship Behavior Scale (Podsakoff et al., 1990, see Paillé, 2006 for a French translation) and the definitions of the different dimensions of CB proposed by Podsakoff et al. (2000), we adapted the measure of CB to the societal (rather than organizational) context. The measure comprised 14 items and was designed to assess four types of behaviors: altruism (e.g., “help people around me, even those I don’t necessarily know”), sportsmanship (e.g., “focus on the positive side, instead of what’s wrong”), civic virtue (e.g., “be willing to participate if the government organizes a public consultation.”) and individual initiative (e.g., “agree to work more if it seems necessary”). Participants were asked to report the extent to which each statement applied to them, on a scale from 1 (*Strongly disagree*) to 7 (*Strongly agree*).

As we developed it for the purpose of the study, we conducted a factor analysis using oblimin direct rotation on the 14 items of CB to examine its structure. The results for both studies are summarized in ***Table 1***. In Study 1, the analysis revealed that four factors had an eigenvalue greater than one, but their content did not exactly match the expected dimensions. The first factor, *altruism*, accounted for 23.66% of the variance and included four items. The second factor, which we labeled *sacrifice for the greater good*, comprised four items and explained an additional 12.91% of the variance. It comprised the two items originally designed for the individual initiative dimension, one item of the sportsmanship and one of the civic virtue dimension (see ***Table 1***). The third factor, *sportsmanship*, included two items of the initial dimension and accounted for 11.16% of the variance. Finally, the fourth factor, *civic virtue*, accounted for 8.32% of the variance and comprised two items of the initial dimension. The analysis conducted in Study 2 revealed a similar structure, with the four factors of altruism, sacrifice, sportsmanship and civic virtue (see ***Table 1***). Because in both studies, the dimensions are sportsmanship (r_S1_ = .31, α_S2_ = .54) and civic virtue (r_S1_ = .12, only one item in Study 2) lacked internal consistency, we decided to focus on the dimensions of altruism (α_S1_ =.78, α_S2_ = .76) and sacrifice (α_S1_=.70, α_S2_= .65) for further analysis.

**Table 1:** Summary of factor analysis results for citizenship behaviors.


	STUDY 1				STUDY 2			
	
	EIGENVALUE	% OF VARIANCE	α (OR r)	FACTOR LOADING	EIGENVALUE	% OF VARIANCE	α (OR r)	FACTOR LOADING

**Factor 1: Altruism**	3.31	23.66	.78		3.58	25.58	.76	

I am willing to help the frailest or most vulnerable people.				.815				.808

I would take care of my relatives if one of them were sick.				.674				.666

I am willing to help people around me, even those I do not necessarily know.				.807				.619

I regularly keep in touch with the isolated people in my relations.				.504				.585

I am willing to take part if the government holds a public consultation.								.320

**Factor 2: Sacrifice for the greater good**	1.81	12.91	.70		1.26	9.00	.65	

I would agree to work more if it is necessary.				.616				.754

I am willing to sacrifice some of my freedom for the good of others.				.556				.569

I am ready to give up my holidays				.692				.547

I try to keep up with all the official statements and speeches given by the Prime Minister, the government or the Royal family.				.500				

**Factor 3: Sportsmanship**	1.56	11.16	.31		1.68	11.97	.54	

I tend to make “mountains out of molehills”. (r)				.565				.754

I have a tendency to focus on the positive side of things, rather than on what is wrong.				–.545				–.441

I always find fault with what the British government does. (r)								.405

**Factor 4: Civic virtue**	1.17	8.32	.12		1.06	7.56		

I discuss the information I think is important on social media (e.g., Twitter).				.468				.687

I am disposed to report behaviour that does not respect the law				.414				


*Note*: Only factor loadings above .30 are shown. For reliability indices, Cronbach’s alpha is reported when the factor comprises at least three items, otherwise, Spearman correlation is reported.

#### Prescriptive gender stereotypes

Participants reported the extent to which they felt that people expected them to show various attitudes and behaviors using a scale from 1 (*Strongly disagree*) to 7 (*Strongly agree*). The 20 items used in the present study correspond to the prescriptions of gender stereotypes for men and women identified by Prentice and Carranza (2002). An example of female prescription (α_S1_ = .77, α_S2_ = .72) is “[people expect me to] be sensitive to the fate of other people” (referring to sensitivity) and an example of male prescription (α_S1_ = .82, α_S2_ = .80) is “make important decisions” (referring to decisiveness).

#### System justification

We assessed participants’ motivation to justify the system using the scale developed by Kay and Jost (2003), which showed great reliability in both studies (α_S1_ = .88; α_S2_= .87). Participants reported the extent to which they agreed with each of the eight statements (e.g., In general, you find society to be fair) using a 7-point scale ranging from 1 (*Strongly disagree*) to 7 (*Strongly agree*).

## Results

### Study 1

#### Manipulation check

We first conducted a between-subject ANOVA on participants’ worry regarding the crisis. The results showed that participants in the high-salience condition (*M* = 59.36, *SE* = 1.77) worried more about the Covid-19 than those in the control condition (*M* = 50.50, *SE* = 2.03), *F*(1,338) = 10.79, *p* = .001, 𝜂*_p_*^2^ = .031.

#### Citizenship behaviors

**Figure 1:**
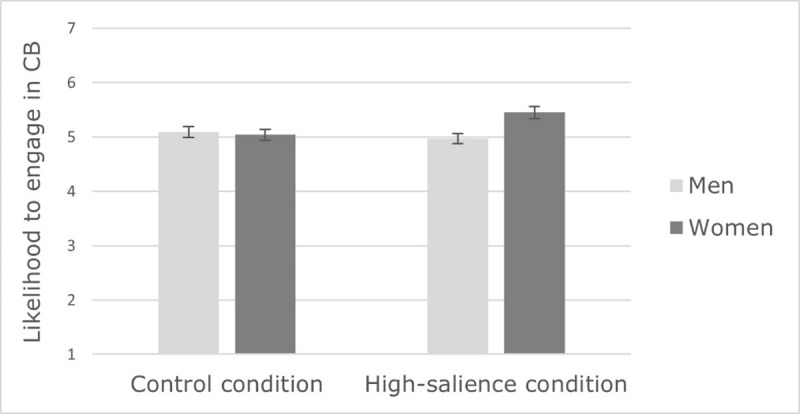
Effect of context on men and women’s likelihood to engage in CB (Study 1).

**Figure 2:**
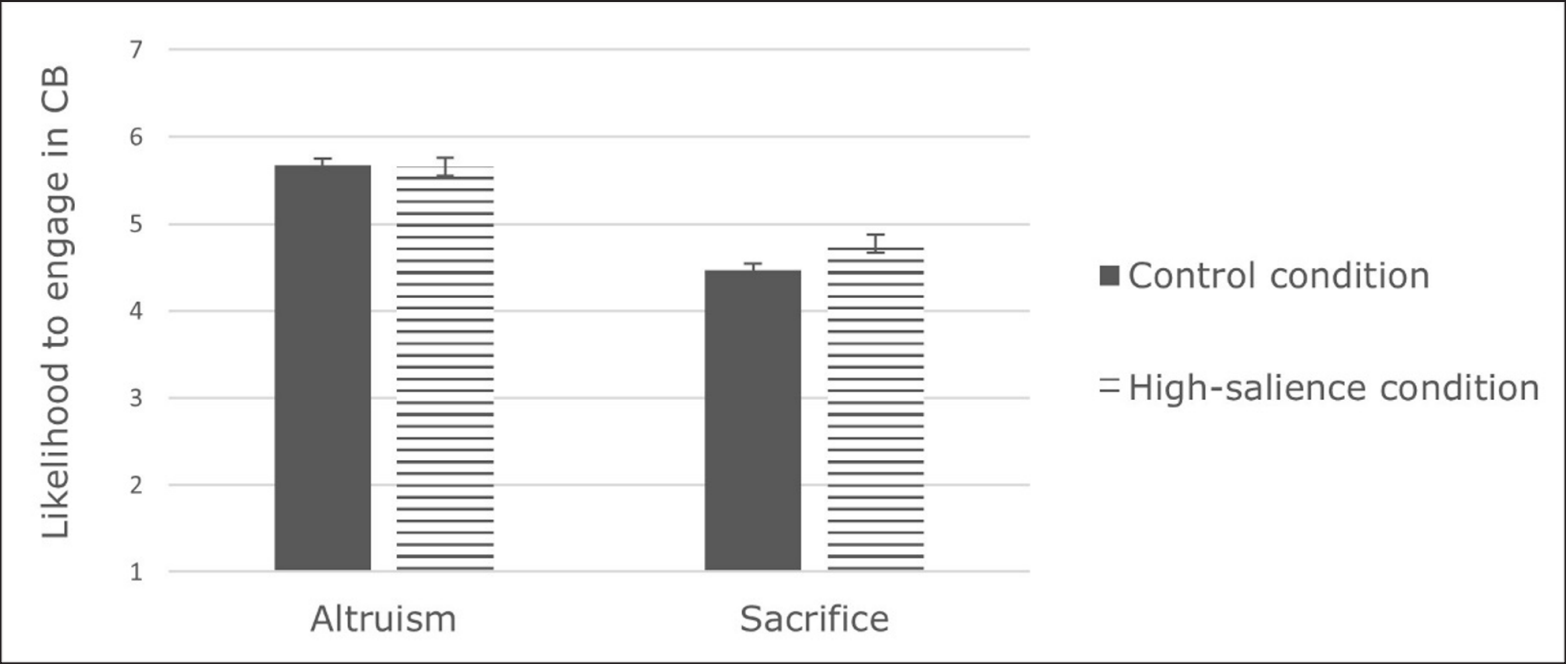
Effect of context on likelihood to engage in CB depending on CB dimension (Study 1).

Following the reliability analyses, only the CB dimensions of altruism and sacrifice were retained for future analyses. We conducted a 2(context: high salience of Covid-19 vs. control, between-subject) × 2(gender: men vs. women) × 2(CB dimension: altruism and sacrifice, within-subject) ANOVA on likelihood to engage in CB. The results revealed a main effect of gender, *F*(1,336) = 4.53, *p* = .034, 𝜂*_p_*^2^ = .013, qualified by a significant interaction with context, *F*(1,336) = 6.69, *p* = .010, 𝜂*_p_*^2^ = .020. Gender differences in CB were significant in the high-salience condition only, *F*(1,336) = 11.07, *p* < .001, 𝜂*_p_*^2^ = .032 (*F* < 1, *ns* for the control condition). More precisely, in the high-salience condition, women were more likely than men to engage in CB of altruism and sacrifice (see ***Figure 1***). In accordance with our hypothesis, women, but not men, are more likely to engage in altruism and sacrifice in the high-salience condition compared to the control condition, *F*(1,336) = 7.23, *p* = .008, 𝜂*_p_*^2^ = .021. The results also indicated a main effect of CB dimension in that participants were more likely to engage in altruism (*M* = 5.66, *SE* = 0.05) than in sacrifice (*M* = 4.62, *SE* = 0.07), *F*(1,336) = 181.69, *p* < .001, 𝜂*_p_*^2^ = .351. This effect was qualified by an interaction with context, *F*(1,336) = 4.14, *p* = .043, 𝜂*_p_*^2^ = .012 (see ***Figure 2***). Participants reported being more likely to engage in CB of altruism than in sacrifice in both the high-salience (*F*(1,336) = 65.15, *p* < .001, 𝜂*_p_*^2^ = .162) and the control condition (*F*(1,336) = 120.97, *p* < .001, 𝜂*_p_*^2^ = .265). However, the context had a significant impact on sacrifice only, *F*(1,336) = 4.30, *p* = .039, 𝜂*_p_*^2^ = .013, in that participants reported more CB of sacrifice, but not of altruism (*F* < 1, *ns)*, in the high salience condition (*M* = 4.77, *SE* = 0.10) than in the control condition (*M* = 4.47, *SE* = 0.10).

#### Prescriptive gender stereotypes

**Figure 3:**
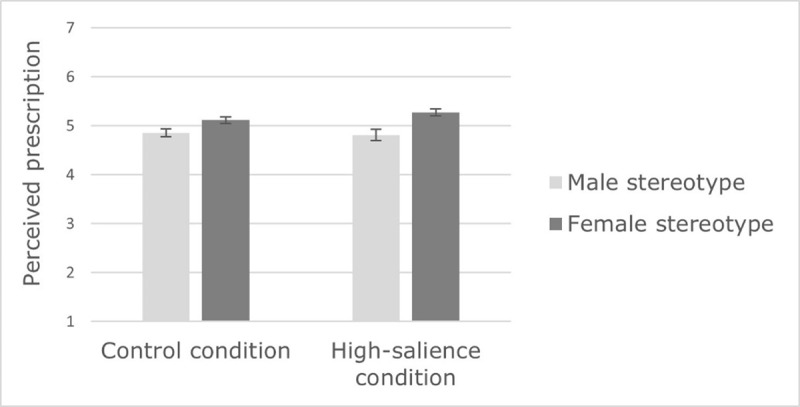
Effect of context on male and female stereotype prescriptions (Study 1).

We ran a 2(context: high salience of Covid-19 vs. control, between-subject) × 2(gender: men vs. women) × 2(stereotype dimension: male and female, within-subject) ANOVA on stereotype prescriptions. The results revealed a main effect of stereotype dimension, *F*(1,336) = 99.10, *p <* .001, 𝜂*_p_*^2^ = .228. Participants perceived the prescriptions related to the female stereotype (*M* = 5.19, *SE* = 0.05) as stronger than those of the male stereotype (*M* = 4.83, *SE* = 0.05). This effect was qualified by a significant interaction with the context, *F*(1,336) = 7.59, *p =* .006, 𝜂*_p_*^2^ = .022. Simple-effect analysis showed that female prescriptions are perceived as stronger than male prescriptions in both conditions, even though greater differences are reported in the high-salience condition (*F*(1,336) = 80.35, *p <* .001, 𝜂*_p_*^2^ = .196) than in the control condition (*F*(1,336) = 26.06, *p <* .001, 𝜂*_p_*^2^ = .072, see ***Figure 3***). Moreover, participants tended to perceive female prescriptions as stronger in the high-salience condition than in the control condition, *F*(1,336) = 2.95, *p =* .087, 𝜂*_p_*^2^ = .009. Context, however, did not affect male prescriptions, *F* < 1, *ns*. There was also a significant interaction between gender and stereotype dimension, *F*(1,336) = 20.79, *p <* .001, 𝜂*_p_*^2^ = .058. Again, simple-effect analysis showed that both men (*F*(1,336) = 16.38, *p <* .001, 𝜂*_p_*^2^ = .046) and women (*F*(1,336) = 94.80, *p <* .001, 𝜂*_p_*^2^ = .220) reported stronger female than male prescriptions. Women (*M* = 5.28, *SE* = 0.07) tended to perceive stronger female prescriptions than men (*M* = 5.11, *SE* = 0.06), *F*(1,336) = 3.31, *p =* .070, 𝜂*_p_*^2^ = .010, whereas there were no significant differences for male prescriptions; *F* < 1, *ns*.

#### System justification

The results revealed no significant effect of either context or gender on participants’ motivation to justify the system, *p* > .10, *ns*.

### Study 2

The analyses are identical to those described in Study 1.

#### Manipulation check

Similar to Study 1, participants in the high-salience condition (*M* = 67.04, *SE* = 1.87) worried more about the Covid-19 than those in the control condition (*M* = 59.67, *SE* = 1.85), *F*(1,321) = 7.86, *p =* .005, 𝜂*_p_*^2^ = .024.

#### Citizenship behaviors

**Figure 4:**
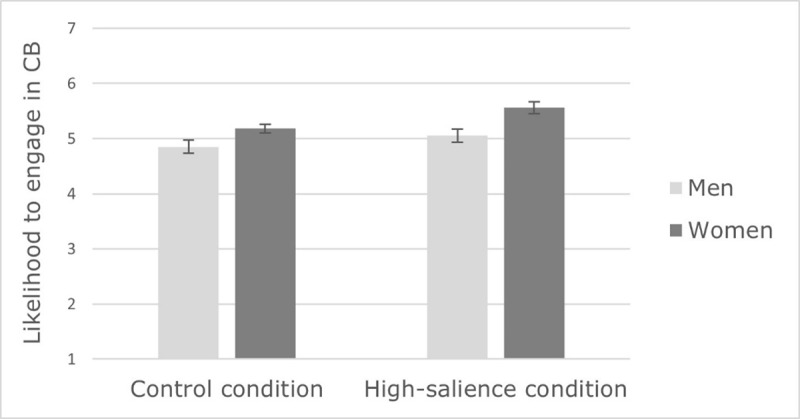
Effect of context on men and women’s likelihood to engage in CB (Study 2).

The results showed a main effect of gender, *F*(1,319) = 17.28, *p* < .001, 𝜂*_p_*^2^ = .051. Women (*M* = 5.37, *SE* = 0.06) were more likely than men (*M* = 4.95, *SE* = 0.08) to engage in CB. A significant main effect of context also indicated that participants were more likely to engage in CB in the high-salience condition (*M* = 5.31, *SE* = 0.07) compared to the control condition (*M* = 5.02, *SE* = 0.07), *F*(1,319) = 8.24, *p* = .004, 𝜂*_p_*^2^ = .025. The interaction between gender and context did not reach significance (*F* < 1, *ns*), as gender differences in CB were significant in both conditions (see ***Figure 4***). However, as in Study 1, context only had a significant effect on women’s (but not men’s) CB (*F_women_* (1,319) = 10.15, *p* = .002, 𝜂*_p_*^2^ = .031; *F_men_*(1,319) = 1.15, *p* = .214, 𝜂*_p_*^2^ = .005). The results revealed a main effect of CB dimension in that participants were more likely to engage in altruism (*M* = 5.54, *SE* = 0.05) than in sacrifice (*M* = 4.78, *SE* = 0.07), *F*(1,319) = 121.25, *p* < .001, 𝜂*_p_*^2^ = .275. Replicating Study 1’s results, this effect was qualified by an interaction with context, *F*(1,319) = 9.66, *p* = .002, 𝜂*_p_*^2^ = .029. Once again, the context had a significant impact of the perceived importance of sacrifice (*M_high-salience_* = 5.03, *SE* = 0.10; *M_control_* = 4.53, *SE* = 0.10), *F*(1,319) = 13.43, *p* < .001, 𝜂*_p_*^2^ = .040, but not on altruism (*F* < 1, *ns)*.

#### Prescriptive gender stereotypes

As in Study 1, the results revealed a main effect of stereotype dimension, *F*(1,319) = 17.40, *p <* .001, 𝜂*_p_*^2^ = .052. Participants perceived the prescriptions related to the female stereotype (*M* = 5.16, *SE* = 0.04) as stronger than those of the male stereotype (*M* = 4.99, *SE* = 0.05). There was also a main effect of gender, *F*(1, 319) = 10.79, *p =* .001, 𝜂*_p_*^2^ = .033, in that women (*M* = 4.94, *SE* = 0.07) reported more pressure to conform to gender stereotypes than men did (*M* = 5.21, *SE* = 0.05), regardless of the dimension. Finally, results showed a marginal main effect of context, *F*(1, 319) = 3.56, *p =* .060, 𝜂*_p_*^2^ = .011. Participants perceived more prescriptions when the Covid-19 crisis was salient (*M* = 5.15, *SE* = 0.06), compared to when it was not (*M* = 5.00, *SE* = 0.06). Contrary to Study 1, neither the interaction between stereotype dimension and context nor its interaction with gender reached significance, *Fs* < 1, *ns*.

#### System justification

Similar to Study 1, the results revealed no significant effect of either context or gender on participants’ motivation to justify the system, *p* > .10, *ns*.

### Mini meta-analysis

Finally, we conducted a mini meta-analysis of Studies 1 and 2 to summarize the results of the two studies. Mini meta-analyses are recommended to improve the replicability of research in psychology and social sciences as they encourage researchers to focus on effect size rather than p-value (reducing the risk of p-hacking and publication bias) and provide a reliable effect size for a phenomenon, a useful information for power calculation in future studies (Cumming, 2014; Goh et al., 2009; Mcshane & Böckenholt, 2017). In the present research, the meta-analysis is even more informative as, based on the p-value of the interaction between context and gender on CB, Study 2 did not perfectly replicate the results of Study 1 (despite consistent simple effects). We therefore computed summary effect sizes for gender differences in CB across studies and tested whether they were larger in the high-salience condition compared to the control condition. We followed Goh et al. (2009)’s guidelines for mini meta-analysis and Borenstein et al. (2009)’s procedure for independent subgroups within studies.

The effect size for gender differences (Cohen’s d) in likelihood to engage in CB in each condition and for each study are presented in ***Table 2***. Because our objective was to compute a common effect size for two identical studies, we computed a summary effect for each condition using a fixed-effect model. The results, reported in ***Table 3***, showed that gender differences were significant in the high-salience condition only. Women were more likely than men to engage in altruism and sacrifice when the Covid-19 crisis was made salient, but not in the control condition. Next, we compared the effect sizes of gender differences of the two conditions using a Z-test. The results are summarized in ***Table 4***. They confirmed that, consistent with our hypothesis, gender differences in altruism were significantly larger in the high-salience condition than in the control condition. Similar trends were observed regarding gender differences in sacrifice, although the differences between conditions were marginally significant.

**Table 2:** Effect sizes (Cohen’s d) for gender differences in CB.


	STUDY 1		STUDY 2	
	
	ALTRUISM	SACRIFICE	ALTRUISM	SACRIFICE

High-salience condition	0.468	0.384	0.521	0.458

Control condition	0.021	–0.083	0.264	0.372


**Table 3:** Summary of meta-analysis results on independent subgroups.


	SUMMARY EFFECT SIZE (d)	SD	Z	p	CONFIDENCE INTERVAL 95%

*High-salience condition*					

Altruism	0.493	0.115	4.279	<.001	[0.267; 0.718]

Sacrifice	0.418	0.115	3.648	<.001	[0.194; 0.643]

*Control condition*					

Altruism	0.134	0.113	1.180	.238	[–0.088; 0.355]

Sacrifice	0.127	0.113	1.119	.263	[–0.095; 0.349]


**Table 4:** Comparison of effect size between independent subgroups.


DEPENDENT VARIABLE	D_diff_	Z_diff_	p

Altruism	0.377	2.339	0.019

Sacrifice	0.304	1.883	0.060


## Discussion

The present research investigates the issue of gender differences in individual responses to the Covid-19 crisis, with a particular focus on CB. Manipulating the salience of the Covid-19 crisis is challenging, as at the time the studies were conducted, the issue was probably already present in the minds of participants due to the considerable impact of the crisis on their daily life. Nevertheless, the analysis of the manipulation check measure shows that participants in both studies feel more worried about the Covid-19 situation in the high-salience condition compared to the control condition. Successfully manipulating the context related to the Covid-19 crisis was important to establish causality. We hypothesized that gender differences in CB would be larger in the high-salience condition than in the control condition. Moreover, because the Covid-19 crisis threatens the system, we also expected that the salience of the crisis would make participants perceive more prescriptions related to the stereotype consistent with their gender (i.e., male prescriptions for men and female prescription for women), compared to when it was not made salient. In Study 1, women – but not men – are more likely to engage in altruism and sacrifice when the Covid-19 crisis is salient (compared to a control condition). This finding is in line with our hypothesis that the Covid-19 would lead people to engage in CB that are consistent with gender stereotypes. Indeed, women report that they are more inclined to show altruism and sacrifice than men are, which supports the assumption of the two dimensions being more consistent with the female rather the male stereotype (Heilman & Chen, 2005). Even though gender does not significantly moderate the effect of context in Study 2, the meta-analysis of the two studies shows that gender differences in altruism and sacrifice are indeed larger in the high-salience condition compared to the control condition, which supports our hypothesis. Contrary to our expectations, the context has no effect on the motivation to justify the system. However, the results indicate a significant effect of context on likelihood to engage in altruistic and sacrifice behaviors, which in our view, could be considered as an indirect support to the system justification hypothesis.

The present research therefore demonstrates that gender can influence individual responses to the Covid-19. Women are ready to help others more and make more sacrifice for the greater good to cope with the situation, which is not the case of men. Women therefore seem more likely to be in charge of caring for others, an especially demanding task in the context of a pandemic. Indeed, women, who were already more likely to take care of domestic and care work before Covid-19, are also more likely to have increased the amount of time dedicated to these tasks since the pandemic began (UN Women, 2020b). Our findings are consistent with literature on gender differences in prosocial behaviors, which shows that, in accordance with gender stereotypes, women are more likely to provide support in close relationships (family, friends, coworkers) and less likely to engage in heroic and/or chivalrous actions and provide collective support than men (Eagly, 2009). Regarding gender stereotypes, results show that men and women in Study 1 report more prescriptions related to female, but not male, stereotype in the high-salience condition than in the control condition. In other words, French participants think people expect them to express more traits and behaviors consistent with female stereotype (e.g., caring, sensitive) in a context in which the Covid-19 crisis is salient than when it is not. These findings, which suggest that feminine stereotypical characteristics (more than masculine ones) might be considered as relevant to deal the Covid-19 crisis, could reflect a “think crisis-think female” association. The “think crisis-think female” association has been identified in the organizational domain with studies showing that people think the ideal manager for a company in times of crisis should have more feminine than masculine traits (Ryan et al., 2011). Indeed, women are more likely to be offered a leadership position in precarious rather than stable situations (Ryan & Haslam, 2005), in part because women are perceived as possessing the characteristics needed to deal with a crisis (Ryan et al., 2016). The intensification of female prescriptions when the Covid-19 crisis is highly salient (vs. control condition) might suggest that the “think crisis-think female” association exists outside of the organizational domain.

### Limitations and perspectives

The present research has some limitations that should be addressed in future research. First, we were not able to identify the expected structure in our measure of CB. Instead, a new form of CB, sacrifice, emerged from the factor analysis. As the present research is the first to apply the concept of CB to society in general, rather than a specific domain (e.g., organization, education), additional research is needed to identify the various dimensions of CB at the societal level. However, the similarities in the factorial structure identified among French (Study 1) and British (Study 2) participants make us confident in the relevance of the dimension of sacrifice for the study of CB in society. Second, the results obtained with the British sample do not fully replicate those obtained with the French sample. To address this limitation, we conducted our mini meta-analysis using a fixed-effect model. If this statistical technique is appropriate for a small number of studies, it does not enable us to generalize our results to other populations (Borenstein et al., 2009). Third, despite the effectiveness of our manipulation, the context does not seem to have a significant impact on participants’ motivation to justify the system in either Study 1, or Study 2. Processes related to system confidence might explain the absence of significant effect of context in our research. Indeed, research has demonstrated that people use different strategies to support the system depending on how much confidence they place in it (Banfield et al., 2011; Cutright et al., 2011; Friesen et al., 2019). When confronted with a system threat, people high in system confidence directly and explicitly defend the system, whereas people low in system confidence tend to choose more indirect forms of support (Cutright et al., 2011). Given the political climate in France (e.g., yellow-vest movement, protest against the retirement reform) and Great Britain (e.g., concretization of the Brexit), we cannot rule out the possibility that our samples might have overall low confidence in their national systems.

## Conclusion

The Covid-19 crisis is a unique, unprecedented situation that evolves very quickly (e.g., French people had less than 24h to prepare to the first lockdown), leading to sudden changes in people’s environment for which the consequences are difficult to anticipate. Psychological research is essential to examine the consequences of such a crisis and to reach a better, more global, understanding of its impact on people. This research, which focuses on individual responses to the crisis, shows that the Covid-19 crisis is associated with gender differences in altruistic and sacrifice behaviors. Women, but not men, are more likely to engage in altruistic behaviors and make sacrifice for the greater good in order to cope with the situation. As such, the crisis may accentuate gender inequalities in society. Indeed, more than ever, women seem to be in charge of caring for others and therefore sanctuarized in their stereotypical gender role by the crisis.

## Footnotes

Study 1’s survey, which included additional exploratory measures (presented in the pre-registration) was twice as long as that of Study 2.

Study 1’s survey, which included additional exploratory measures (presented in the pre-registration) was twice as long as that of Study 2.

## Author Informations

**Alyson Sicard** is a post-doctoral researcher at the University Clermont Auvergne as part of the DISESTEEM project, and a member of the LASPCO (Laboratoire de Psychologie Sociale et Cognitive). Her research interests include gender inequalities and the consequences of threatening academic contexts.

**Delphine Martinot** is a Professor of Social Psychology in the Laboratoire de Psychologie Sociale et Cognitive UMR CNRS 6024 of University Clermont Auvergne. Her research focuses on gender stereotypes, school achievement, and academic disengagement. She studies how to decrease academic inequalities, especially among female and male pupils and students.

**Céline Darnon** is a professor of social psychology at Université Clermont Auvergne, France. Her research examines how the educational system, because of the values and the goals it promotes, contribute to legitimate and reproduce class and gender inequalities.

**Sandrine Redersdorff** is senior lecturer in Social Psychology in the Laboratoire de Psychologie Sociale et Cognitive UMR CNRS 6024 of University Clermont Auvergne. Her research focuses on social inequalities and the self-esteem protection strategies among disadvantaged groups. She also investigates social costs of denouncing sexism, gender discrimination and glass cliff effect.

## References

[1] Al Dhaheri, A. S., Bataineh, M. F., Mohamad, M. N., Ajab, A., Al Marzouqi, A., Jarrar, A. H., Habib-Mourad, C., Jamous, D. O. A., Ali, H. I., Al Sabbah, H., Hasan, H., Stojanovska, L., Hashim, M., Elhameed, O. A. A., Obaid, R. R. S., ElFeky, S., Saleh, S. T., Osaili, T. M., & Ismail, L. C. (2021). Impact of COVID-19 on mental health and quality of life: Is there any effect? A crosssectional study of the MENA region. PLoS ONE, 16(3), 1–17. DOI: 10.1371/journal.pone.0249107 PMC7993788 33765015

[2] Allen, T. D., & Jang, R. (2018). Gender and organizational citizenship behavior. In P. M. Podsakoff, S. B. MacKenzie, & N. P. Podsakoff (Eds.), The Oxford Handbook of Organizational Citizenship Behavior. DOI: 10.1093/oxfordhb/9780190219000.013.12

[3] Aoyagi, M. W., Cox, R. H., & McGuire, R. T. (2008). Organizational citizenship behavior in sport: Relationships with leadership, team cohesion, and athlete satisfaction. Journal of Applied Sport Psychology, 20(1), 25–41. DOI: 10.1080/10413200701784858

[4] Asendorpf, J. B., Conner, M., De Fruyt, F., De Houwer, J., Denissen, J. J. A., Fiedler, K., Fiedler, S., Funder, D. C., Kliegl, R., Nosek, B. A., Perugini, M., Roberts, B. W., Schmitt, M., Van Aken, M. A. G., Weber, H., & Wicherts, J. M. (2013). Recommendations for increasing replicability in psychology. European Journal of Personality, 27(2), 108–119. DOI: 10.1002/per.1919

[5] Banfield, J. C., Kay, A. C., Cutright, K. M., Wu, E. C., & Fitzsimons, G. J. (2011). A person by situation account of motivated system defense. Social Psychological and Personality Science, 2(2), 212–219. DOI: 10.1177/1948550610386809

[6] Bonnot, V., & Jost, J. T. (2014). Divergent effects of system justification salience on the academic self-assessments of men and women. Group Processes & Intergroup Relations, 17(4), 453–464. DOI: 10.1177/1368430213512008

[7] Borenstein, M., Hedges, L., Higgins, J., & Rothstein, H. R. (2009). Introduction to Meta-Analysis. DOI: 10.1002/9780470743386

[8] Brandt, M. J., IJzerman, H., Dijksterhuis, A., Farach, F. J., Geller, J., Giner-Sorolla, R., Grange, J. A., Perugini, M., Spies, J. R., & van ’t Veer, A. (2014). The Replication Recipe: What makes for a convincing replication? Journal of Experimental Social Psychology, 50(1), 217–224. DOI: 10.1016/j.jesp.2013.10.005

[9] Brescoll, V. L., Uhlmann, E. L., & Newman, G. E. (2013). The effects of system-justifying motives on endorsement of essentialist explanations for gender differences. Journal of Personality and Social Psychology, 105(6), 891–908. DOI: 10.1037/a0034701 24295379

[10] Chiaburu, D. S., Harris, T. B., & Smith, T. A. (2014). Ideology and gender: Observers system justification and targets gender as interactive predictors of citizenship expectations. Journal of Social Psychology, 154(4), 283–298. DOI: 10.1080/00224545.2014.893975 25154113

[11] Chiaburu, D. S., Sawyer, K., Smith, T. A., Brown, N., & Harris, T. B. (2014). When Civic Virtue isn’t Seen as Virtuous: The Effect of Gender Stereotyping on Civic Virtue Expectations for Women. Sex Roles, 70(5–6), 183–194. DOI: 10.1007/s11199-014-0346-z

[12] Clarke, H. M., & Sulsky, L. M. (2017). The impact of gender ideology on the performance of gender-congruent citizenship behaviors. Human Performance, 30(4), 212–230. DOI: 10.1080/08959285.2017.1361958

[13] Cumming, G. (2014). The New Statistics. Psychological Science, 25(1), 7–29. DOI: 10.1177/0956797613504966 24220629

[14] Cutright, K. M., Wu, E. C., Banfield, J. C., Kay, A. C., & Fitzsimons, G. J. (2011). When Your World Must Be Defended: Choosing Products to Justify the System. Journal of Consumer Research, 38(1), 62–77. DOI: 10.1086/658469

[15] Eagly, A. H. (2009). The His and Hers of Prosocial Behavior: An Examination of the Social Psychology of Gender. American Psychologist, 64(8), 644–658. DOI: 10.1037/0003-066X.64.8.644 19899859

[16] Friesen, J. P., Laurin, K., Shepherd, S., Gaucher, D., & Kay, A. C. (2019). System justification: Experimental evidence, its contextual nature, and implications for social change. British Journal of Social Psychology, 58(2), 315–339. DOI: 10.1111/bjso.12278 30229936

[17] Goh, J. X., Hall, J. A., & Rosenthal, R. (2009). Mini Meta-Analysis of Your Own Studies: Some Arguments on Why and a Primer on How. Social and Personality Psychology Compass, 10(10), 535–549. DOI: 10.1111/spc3.12267

[18] Goodwin, R., Wiwattanapantuwong, J., Tuicomepee, A., Suttiwan, P., Watakakosol, R., & Ben-Ezra, M. (2021). Anxiety, perceived control and pandemic behaviour in Thailand during COVID-19: Results from a national survey. Journal of Psychiatric Research, 135, 212–217. DOI: 10.1016/j.jpsychires.2021.01.025 33497875 PMC7826082

[19] Heilman, M. E., & Chen, J. J. (2005). Same behavior, different consequences: Reactions to men’s and women’s altruistic citizenship behavior. Journal of Applied Psychology, 90(3), 431–441. DOI: 10.1037/0021-9010.90.3.431 15910140

[20] Jost, J. T. (2019). A quarter century of system justification theory: Questions, answers, criticisms, and societal applications. British Journal of Social Psychology, 58(2), 263–314. DOI: 10.1111/bjso.12297

[21] Jost, J. T., & Banaji, M. R. (1994). The Role of Stereotyping in System-justification and the Production of False Consciousness. British Journal of Social Psychology, 33, 1–27. DOI: 10.1111/j.2044-8309.1994.tb01008.x

[22] Jost, J. T., & Hunyady, O. (2005). Antecedents and Consequences of System-Justifying Ideologies. Current Directions in Psychological Science, 14(5), 114–118. DOI: 10.1111/j.0963-7214.2005.00377.x

[23] Kay, A. C., & Friesen, J. (2011). On Social Stability and Social Change: Understanding When System Justification Does and Does Not Occur. Current Directions in Psychological Science, 20(6), 360–364. DOI: 10.1177/0963721411422059

[24] Kay, A. C., & Jost, J. T. (2003). Complementary justice: Effects of “poor but happy” and “poor but honest” stereotype exemplars on system justification and implicit activation of the justice motive. Journal of Personality and Social Psychology, 85(5), 823–837. DOI: 10.1037/0022-3514.85.5.823 14599247

[25] Kidder, D. L. (2002). The influence of gender on the performance of organizational citizenship behaviors. Journal of Management, 28(5), 629–648. DOI: 10.1016/S0149-2063(02)00159-9

[26] Laurin, K., Kay, A. C., & Shepherd, S. (2011). Self-Stereotyping as a Route to System Justification. Social Cognition, 29(3), 360–375. DOI: 10.1521/soco.2011.29.3.360

[27] Lepine, J. A., Erez, A., & Johnson, D. E. (2002). The nature and dimensionality of organizational citizenship behavior: a critical review and meta-analysis. The Journal of Applied Psychology, 87(1), 52–65. DOI: 10.1037/0021-9010.87.1.52 11916216

[28] Mcshane, B. B., & Böckenholt, U. (2017). Single Paper Meta-analysis: Benefits for Study Summary, Theory-testing, and Replicability. Journal of Consumer Research, 43, ucw085. DOI: 10.1093/jcr/ucw085

[29] Meriac, J. P. (2012). Work ethic and academic performance: Predicting citizenship and counterproductive behavior. Learning and Individual Differences, 22(4), 549–553. DOI: 10.1016/j.lindif.2012.03.015

[30] Organ, D. W. (1998). Organizational citizenship behavior: The good soldier syndrome. Lexington Books/DC Heath and Com.

[31] Paillé, P. (2006). Les relations entre l’implication au travail, les comportements de citoyenneté organisationnelle et l’intention de retrait. Revue Europeenne de Psychologie Appliquee, 56(2), 139–149. DOI: 10.1016/j.erap.2005.06.001

[32] Podsakoff, P. M., MacKenzie, S. B., Moorman, R. H., & Fetter, R. (1990). Transformational leader behaviors and their effects on followers’ trust in leader, satisfaction, and organizational citizenship behaviors. The Leadership Quarterly, 1(2), 107–142. DOI: 10.1016/1048-9843(90)90009-7

[33] Podsakoff, P. M., MacKenzie, S. B., Paine, J. B., & Bachrach, D. G. (2000). Organizational citizenship behaviors: A critical review of the theoretical and empirical literature and suggestions for future research. Journal of Management, 26(3), 513–563. DOI: 10.1177/014920630002600307

[34] Prentice, D. A., & Carranza, E. (2002). What Women and Men Should Be, Shouldn’t Be, Are Allowed to Be, and Don’t Have to Be: The Contents of Prescriptive Gender Stereotypes. Psychology of Women Quarterly, 26(4), 269–281. DOI: 10.1111/1471-6402.t01-1-00066

[35] Ryan, M. K., & Haslam, S. A. (2005). The glass cliff: Evidence that women are over-represented in precarious leadership positions. British Journal of Management, 16(2), 81–90. DOI: 10.1111/j.1467-8551.2005.00433.x

[36] Ryan, M. K., Haslam, S. A., Hersby, M. D., & Bongiorno, R. (2011). Think crisis-think female: The glass cliff and contextual variation in the think manager-think male stereotype. Journal of Applied Psychology, 96(3), 470–484. DOI: 10.1037/a0022133 21171729

[37] Ryan, M. K., Haslam, S. A., Morgenroth, T., Rink, F., Stoker, J., & Peters, K. (2016). Getting on top of the glass cliff: Reviewing a decade of evidence, Explanations, And impact. Leadership Quarterly, 27(3), 446–455. DOI: 10.1016/j.leaqua.2015.10.008

[38] Świątkowski, W., & Dompnier, B. (2017). Replicability Crisis in Social Psychology: Looking at the Past to Find New Pathways for the Future. International Review of Social Psychology, 30, 111–124. DOI: 10.1017/CBO9780511752223

[39] UN Women. (2020a). COVID-19 and ending violence against women and girls. In United Nations. https://www.unwomen.org/-/media/headquarters/attachments/sections/library/publications/2020/issue-brief-covid-19-and-ending-violence-against-women-and-girls-en.pdf?la=en&vs=5006

[40] UN Women. (2020b). Whose time to care? Unpaid care and domestic work during Covid-19. https://data.unwomen.org/publications/whose-time-care-unpaid-care-and-domestic-work-during-covid-19

[41] UNFPA. (2020). Covid-19: A Gender Lens. Protecting sexual and reproductive health and rights, and promoting gender equality.

[42] United Nations Department of Global Communications. (2020). Gender equality in the time of COVID-19. https://www.un.org/en/un-coronavirus-communications-team/gender-equality-time-covid-19

[43] Xiong, J., Lipsitz, O., Nasri, F., Lui, L. M. W., Gill, H., Phan, L., Chen-Li, D., Iacobucci, M., Ho, R., Majeed, A., & McIntyre, R. S. (2020). Impact of COVID-19 pandemic on mental health in the general population: A systematic review. Journal of Affective Disorders, 277(July), 55–64. DOI: 10.1016/j.jad.2020.08.001 32799105 PMC7413844

